# Sanitation, antibiotics, and the end of the antibiotic era

**DOI:** 10.1016/j.puhip.2026.100839

**Published:** 2026-07-29

**Authors:** Michael Weinbren, Susanne Surman-Lee

**Affiliations:** a New Hospital Programme, NHS E, Wellington House, London, England, WC2R 0AP, UK; b Leegionella Ltd, Ringwood, UK

**Keywords:** Antimicrobial resistance, Sanitation, Wastewater, Antimicrobial stewardship

## Abstract

A decade after the publication of the highly influential O'Neill report, *Tackling Drug-Resistant Infections Globally*, antimicrobial resistance (AMR) continues to accelerate at an unprecedented rate. Despite being a cornerstone of global AMR strategies, antimicrobial stewardship has failed to reverse this trend. This failure reflects a fundamental misunderstanding of AMR as primarily a behavioural or prescribing problem, rather than an environmental one.

A major driver of AMR is environmental pollution arising from the large-scale manufacture, release, and accumulation of antimicrobial substances that profoundly disrupt microbial ecosystems. While regulatory attention has focused on antibiotic contamination of local rivers during pharmaceutical manufacturing, this represents only a small fraction of the antibiotics produced and consumed globally. Following administration, and depending on the compound, up to 90% of biologically active antibiotic may be excreted unchanged and subsequently enter healthcare wastewater systems.

Healthcare wastewater systems therefore function as a superhighway for the generation, amplification, and transmission of multidrug-resistant organisms—both within healthcare facilities and into surrounding municipal infrastructure. Traditional hospital defences, including Standard Infection Control Precautions, offer limited protection against pathogens originating from wastewater environments. Within these systems, high microbial biomass is continuously exposed to complex mixtures of antimicrobials, disinfectants, and other selective agents, distorting microbial ecology and creating conditions conducive to the selection of AMR.

Recognising the healthcare wastewater system as a critical driver of AMR generation and dissemination reframes the problem and opens new opportunities for intervention. Addressing this overlooked reservoir may be essential to containing a global pandemic that, despite decades of effort, shows no sign of slowing.

## 1 Introduction

The foundations of modern public health include safe water and effective sanitation. The importance of these developments was underlined by a poll of more than 11,300 readers of the *British Medical Journal* in 2007 [1], who selected safe water and sanitation as the most important medical advance since 1840. Many of the forefathers of modern public health—such as John Snow, Edwin Chadwick, and Joseph Bazalgette—were intimately involved in both elucidating the risks associated with poor sanitation and developing the mitigations that ultimately controlled the cholera pandemic.

The second choice in the BMJ poll was antibiotics. Nearly 20 years later, the world is facing another pandemic of major public health importance: the end of the antibiotic era. The predicted impact of this pandemic is profound, with projected estimates of more than $100 trillion in global economic costs and 10 million excess deaths per year. Antimicrobial resistance (AMR) is projected to become the leading cause of mortality worldwide [2], threatening the future of many modern medical interventions that depend upon effective antimicrobial agents to protect patients at their most vulnerable. Without effective antibiotics, many procedures will either carry unacceptably high mortality or will no longer be practised because they offer no net improvement in outcomes.

Paradoxically, it may be the interplay between sanitation and antibiotics that is both driving the end of the antibiotic era and exposing deficiencies in our sanitation systems. While the design of sanitation systems was effective in controlling the cholera pandemic, do these systems now require updating in light of the AMR pandemic? The evidence to support this proposition is strong and forms the central argument of this article.

## 2 The end of the antibiotic era

For an increasing number of UK patients, the end of the antibiotic era has already arrived. Microorganisms resistant to all known antibiotics (pan-resistant organisms) are now present in some UK hospitals and causing infections. Even where one or two effective agents remain, infections caused by multidrug-resistant organisms (MDROs) carry a high mortality. This is largely because administration of effective therapy is frequently delayed.

When a patient develops a severe infection such as a bloodstream infection (BSI), blood cultures are taken and empirical antimicrobial therapy is initiated while awaiting results. Patients who receive ineffective empirical therapy, as determined by laboratory susceptibility testing, experience substantially higher mortality. In a recent large multicentre study across the USA, 20% of patients with BSI were receiving ineffective empirical therapy. In contrast, a UK study found that 80% of patients with MDRO bloodstream infections were on ineffective treatment [3].

Antimicrobial resistance would be manageable if bacteria were resistant to only one or two classes of antimicrobial agents. The crisis arises when organisms acquire resistance to the vast majority, if not all, available antimicrobials, and in addition possess resistance determinants to biocides and heavy metals through linked resistance genes on mobile genetic elements [4,5]. Darwinian principles dictate that such organisms require an environment applying sustained and diverse selective pressures in order to emerge and persist.

## 3 Antimicrobial stewardship

The term *stewardship* implies taking care of something. Antimicrobials are most powerful when contained; once released into the environment through administration, they resemble a genie let out of the bottle, with unintended and often irreversible consequences.

Humans have taken substances that largely occur naturally in the environment and industrialised their production, delivering quantities of antibiotics that exceed by orders of magnitude anything found in nature. Antimicrobial resistance is, at its core, a consequence of antibiotic pollution of the environment, producing profound effects on local microbiomes. It is widely acknowledged that when a broad-spectrum antibiotic is administered to treat a specific pathogen, extensive collateral damage occurs within the human microbiome. This can result in antibiotic-associated diarrhoea, *Clostridioides difficile* infection, and overgrowth of resistant organisms.

Antimicrobial stewardship has therefore become a cornerstone in the fight against AMR. In 2024, the World Health Organization published new global guidance aimed at curbing antibiotic pollution from manufacturing, although this appears to represent a small proportion of total emissions compared with post-consumption excretion [6]. Following manufacture and distribution, stewardship efforts should focus on preventing unnecessary antibiotic use and, where treatment is required, selecting the narrowest effective agent for the shortest possible duration.

More recently, the World Health Assembly adopted an updated Global Action Plan on Antimicrobial Resistance (2026–2036) in May 2026 [7]. Post-consumption excretion into healthcare wastewater—the specific mechanism this article addresses is not included in the plan or the subject of separate guidance.

Antimicrobial stewardship within healthcare premises has developed into a substantial discipline. While significant reductions in antibiotic usage have been achieved, this has not halted the spread of AMR. Indeed, dissemination appears to be accelerating especially as current stewardship frameworks fail to acknowledge the greatest source of antibiotic pollution; human excretion. For example, two last-resort antibiotics—ceftazidime–avibactam and cefiderocol—are excreted largely unchanged (≥90% biologically active) via the kidneys [[8], [9], [10]]. Consequently, the majority of the world's supply of these critical antibiotics is discharged directly into healthcare wastewater systems and onwards.

Continuing with the same strategies to combat AMR is unlikely to yield a different outcome; so a fundamentally different approach is required.

Healthcare facility sanitation and wastewater systems provide an ideal environment for microorganisms to acquire resistance to antibiotics, biocides, and heavy metals but simultaneously offer unique opportunities to curb the AMR pandemic.

## 4 Dame Sally Davies and Lord Jim O'Neill

Dame Sally Davies, former UK Chief Medical Officer, is widely credited with elevating AMR to the global stage. This was followed by the highly influential O'Neill Review, *Tackling Drug-Resistant Infections Globally*, which provided nations with a blueprint for action. The report was instrumental in galvanising international efforts and remains a landmark contribution.

However, a decade later, new evidence has emerged that challenges some of the assumptions underpinning the original recommendations, particularly in relation to healthcare premises. Sanitation was largely framed as an issue affecting low- and middle-income countries. This is no longer tenable. Even in the most sophisticated healthcare facilities, there is good evidence that wastewater organisms are being transmitted to patients and causing HCAIs.

The key healthcare-focused components of national AMR strategies were infection control, antimicrobial stewardship, and, in the long term, the development of new antibiotics. Although there is the potential for new classes of antibiotics in the pipeline there remains ‘a pressing need for new, innovative agents for serious infections and to replace those becoming ineffective due to widespread use’ [11]. As discussed, stewardship alone is insufficient. This leaves infection control.

While robust infection prevention practices are essential, it has become clear that Standard Infection Control Precautions (SICPs), designed as a universal protective approach for both patients and staff, are largely ineffective in preventing transmission of water- and wastewater-associated organisms [12]. This represents a critical gap in hospital defences. The built environment not only acts as a reservoir for organisms but also provides transmission pathways that are not readily controlled by SICPs.

Surveillance, intended as a final safety net, also lacks sensitivity for detecting transmission events originating from water and wastewater sources. Ironically, the emergence of MDROs has drawn attention to these pathways because resistant organisms are more readily detected. However, antimicrobial-susceptible organisms have likely been exploiting these routes for decades, remaining largely invisible.

## 5 Hospital sanitation and wastewater systems

Hospital sanitation and wastewater systems must be considered from three perspectives:

1. The sanitation/wastewater system as an environment
2. Transmission within the healthcare setting
3. Transmission beyond the healthcare setting

## 6 The sanitation/wastewater environment

Wastewater systems should no longer be viewed as inanimate infrastructure but as complex, living ecosystems with rich microbiomes. While significant efforts are made to limit biofilm formation in potable water systems, this is not the case for sanitation systems. By design, sanitation systems contain abundant nutrients, favourable temperatures, and extensive surfaces that promote biofilm formation.

Many MDROs that threaten the antibiotic era are carried in the human gut and therefore enter wastewater systems in large numbers. The materials used in sanitation infrastructure are not designed to inhibit microbial growth, effectively creating vast surfaces for biofilm development.

The sanitation system has been described as a “superhighway” for microbial movement within healthcare facilities [13]. Organisms can ascend vertical sewage stacks via aerosols generated when air is displaced by wastewater flow. This mechanism is thought to have contributed to the SARS outbreak in Hong Kong and has since been experimentally confirmed using tracer organisms [14]. Microorganisms can also migrate in horizontal pipework between waste traps and even against gravity within interconnected pipework [15].

Studies have demonstrated that organisms placed in a clean waste trap attached to a sink are transformed when a carbon source is added. The organisms then grow up vertical sections of the pipework at a rate of 1 mm/h to reach the sieve in the sink, where they may be dispersed into the room environment by water from an outlet hitting the drain.

Analysis of municipal wastewater consistently shows that healthcare facilities are the dominant contributors to AMR, by orders of magnitude [16]. Although antibiotics are used in the community, they are typically narrower-spectrum and used for shorter durations.

Sampling hospital sewage stacks has demonstrated sustained concentrations of antibiotics such as meropenem at levels exceeding the minimum inhibitory concentration for some organisms throughout the day [17]. Resistance patterns vary within hospitals, correlating closely with antibiotic usage on specific wards.

It should therefore come as no surprise that the most resistant organisms have taken up residence in the very buildings where the most vulnerable patients in society are treated, healthcare premises.

## 7 Transmission within the healthcare setting

The severity of this issue is illustrated by the emergence of “water-free” care units, first described by Joost Hopman and colleagues in 2017 [18]. While the term is misleading, it originally referred to the removal of clinical handwash sinks from individual patient rooms. This intervention was implemented to control an otherwise intractable MDRO outbreak originating from sink wastewater systems. The term water and wastewater safe environments is more accurate.

Since then, water-free designs have been adopted worldwide, including in neonatal intensive care units and single-room facilities. In April 2025, the first UK water-free unit opened at Wexham Hospital. These interventions have not only reduced MDRO transmission but also decreased infections caused by antibiotic-susceptible Gram-negative organisms (it is still early days at Wexham Park and the situation is being continuously monitored, but in the first nine months this is the case – personal communication Manjula Meda).

Transmission events linked to water and wastewater occur throughout healthcare facilities, not only in patient rooms [19,20]. Routine activities such as bed bathing can result in contamination when clean pulp bowls contact sink drains. Similarly, patient water jugs are often placed in sinks, allowing wastewater organisms to contaminate drinking vessels.

The design of shower drains allows patients to come into direct contact with water from drains, contaminating the shower floor, as well as from splashing and aerosolisation, facilitating MDRO spread. In one hospital-wide MDRO outbreak, the source was traced to the main kitchen, likely due to splash-back from sink drains or high-pressure hosing of floor drains. Ward-level decontamination of equipment in sinks further facilitates transmission.

At present, there is no truly safe design for clinical handwash stations that addresses these risks. In the UK sinks with a rear drain are preferred to those with the drain on the floor of the sink, as the risk of dispersal of wastewater organisms is much lower. However, the Achilles heel of the rear drain is that there is no sieve (plastic sieves are available but are thought to cause more problems than they solve as they provide a surface for biofilm formation) to prevent items falling down into the waste trap. Once free flowing drainage is impaired, dispersal of wastewater organisms can occur [21]. Additionally clinical hand wash stations are subject to the vagaries of human interaction-although they should be used for only one purpose, hand decontamination a study using a video camera mounted above a clinical hand wash station on a critical care unit revealed that only 4% of visits were for this purpose [22]. In other words 96 out of every hundred visits were for the wrong purpose [21]. Solving this problem will require a multidisciplinary effort involving clinicians, engineers, infection prevention specialists, and manufacturers.

## 8 Transmission beyond the healthcare setting

Hospital wastewater enters municipal sewage systems and, in high-income countries, is usually treated before discharge. However, wastewater treatment plants do not fully eliminate antibiotic resistance determinants, allowing resistant organisms to enter surface waters. During periods of heavy rainfall, combined sewer overflows may discharge untreated sewage directly into the environment, with documented links to human infection.

In low- and middle-income countries, wastewater is sometimes used for irrigation of crops, including leafy vegetables, creating a direct pathway for reintroduction of AMR into human populations.

## 9 Potential solutions

Several strategies could mitigate risks from hospital wastewater systems:

1. **Preventing antibiotic pollution at source**The largest source of antibiotic pollution is from patient excretion. Waterless toilets (the key being that these are not connected to the wastewater system), already used in both low- and high-income settings (e.g. festivals), collect human waste for controlled disposal (rather than dilution into wastewater/sewage systems). The driver for these developments includes the growing problem of global water shortages [23]. Given the scale of the AMR crisis, such systems should be urgently evaluated, refined, and assessed for acceptability in healthcare settings. There are likely to be a range of options to mitigate this issue, but currently as the risk is not recognised it has not been brought to the attention of individuals with the requisite expertise including engineers/manufacturers who could develop solutions. Life-cycle cost and environmental assessments of resource-oriented toilet systems indicate that designs recovering nutrients from urine alone can achieve a more favourable economic and environmental balance than conventional water-based toilets, and that fully independent systems are feasible in settings lacking sewer and water infrastructure [24].
2. **Wastewater bioreactors**Bioreactors placed between hospital and municipal wastewater systems can remove antibiotics, resistance genes, and other toxic compounds [25]. Despite being developed in the 1990s and commercially available in the new millennium their application and use to remove the toxicity from hospital wastewater is uncommon and restricted to a few countries who have instituted widespread use [26]. Some hospitals in the Netherlands have already implemented such systems. Their value extends beyond AMR, given the unknown long-term effects of many healthcare-derived pollutants.
3. **Expansion of water-free (water and wastewater safe environments) care models**The evidence base for water-free care, while still emerging, is growing—and where it exists, the effect sizes are substantial. A 2024 systematic review identified seven quasi-experimental studies, spanning 332 ICU beds, that used sink removal—with or without complementary water-free measures such as water-less bath products, bottled water, or a designated ‘contaminated’ sink for wastewater disposal—to control water-borne healthcare-associated infections. Five of the seven studies (71.4%) reported cessation of their outbreak, and across units, pathogen incidence densities fell significantly, by 1.2- to 4.2-fold; in one study [27], The principles of water-free care should be further developed and implemented across healthcare facilities. This requires collaboration between infection prevention specialists with expertise in the built environment and frontline clinical teams. There are several challenges – at first sight the removal of clinical hand wash stations to improve patient safety appears counterintuitive. Secondly many infection prevention and control (IPC) specialists, through no fault of their own have received no training in the built environment which is a rapidly emerging discipline. Training both for clinical teams and IPC is therefore key to delivery, and ultimately clinical teams need to have the confidence and ownership through development of the necessary mitigations required to improve and protect patient safety. Innovations such as the ‘Frimley Faucet’ which mitigate the risk of patient water jugs and clean pulp product bowls making contact with the drain whilst being filled and then becoming a vehicle for transmission of wastewater organisms have been developed [28]. The cost of such interventions varies depending on whether this is a refurbishment or a new build. For new build hospitals, incorporation of water and wastewater safe environments will save money both in terms of capital expenditure, ongoing maintenance costs when the hospital opens and reduced transmission of infections and AMR from water and wastewater systems which are not only in themselves costly but incur a significant morbidity and mortality. For refurbishments (most of the NHS estate is legacy estate) this will require some pump priming money but again will prove to be cost-effective as this reduces ongoing maintenance costs and transmission of infection.

## 10 Summary

Safe water and sanitation were among the greatest public health achievements in history, helping to control pandemics such as cholera. The current AMR pandemic is exposing fundamental deficiencies in sanitation systems, particularly within hospitals, where these systems both drive resistance and facilitate transmission.

Despite growing evidence, learning about the risks posed by healthcare wastewater systems has not yet translated into sufficient action. Public health has a crucial role to play in shaping a coordinated national response—driving mitigation strategies, increasing awareness, and engaging society in addressing what is otherwise an unstoppable force.

## Ethics statement

Ethical approval is not necessary for this type of paper, as it is not a research paper, involves no human participants, patient data, or new primary research.

## Funding

None.

## Conflict of interest

This is to state we have no conflict of interest and that we have received no funding.
